# Whole-Genome Sequencing for the Investigation of a Hospital Outbreak of MRSA in China

**DOI:** 10.1371/journal.pone.0149844

**Published:** 2016-03-07

**Authors:** Zhenzhen Kong, Peipei Zhao, Haibing Liu, Xiang Yu, Yanyan Qin, Zhaoliang Su, Shengjun Wang, Huaxi Xu, Jianguo Chen

**Affiliations:** 1 Department of Clinical Laboratory, The Affiliated People’s Hospital of Jiangsu University, Zhenjiang, China; 2 Department of Immunology, Institute of Laboratory Medicine, Jiangsu University, Zhenjiang, China; 3 Department of Neurosurgery, The Affiliated People’s Hospital of Jiangsu University, Zhenjiang, PR China; Rockefeller University, UNITED STATES

## Abstract

*Staphylococcus aureus* is a globally disseminated drug-resistant bacterial species. It remains a leading cause of hospital-acquired infection, primarily among immunocompromised patients. In 2012, the Affiliated People’s Hospital of Jiangsu University experienced a putative outbreak of methicillin-resistant *S*. *aureus* (MRSA) that affected 12 patients in the Neurosurgery Department. In this study, whole-genome sequencing (WGS) was used to gain insight into the epidemiology of the outbreak caused by MRSA, and traditional bacterial genotyping approaches were also applied to provide supportive evidence for WGS. We sequenced the DNA from 6 isolates associated with the outbreak. Phylogenetic analysis was constructed by comparing single-nucleotide polymorphisms (SNPs) in the core genome of 6 isolates in the present study and another 3 referenced isolates from GenBank. Of the 6 MRSA sequences in the current study, 5 belonged to the same group, clustering with T0131, while the other one clustered closely with TW20. All of the isolates were identified as ST239-SCC*mec*III clones. Whole-genome analysis revealed that four of the outbreak isolates were more tightly clustered into a group and SA13002 together with SA13009 were distinct from the outbreak strains, which were considered non-outbreak strains. Based on the sequencing results, the antibiotic-resistance gene status (present or absent) was almost perfectly concordant with the results of phenotypic susceptibility testing. Various toxin genes were also analyzed successfully. Our analysis demonstrates that using traditional molecular methods and WGS can facilitate the identification of outbreaks and help to control nosocomial transmission.

## Introduction

*Staphylococcus aureus* is a leading cause of healthcare-associated infection and remains a globally important human pathogen. The ST239 lineage of methicillin-resistant *S*. *aureus* (MRSA), which has caused multiple epidemics in recent decades around the world, is one of the most widely disseminated hospital-associated MRSA (HA-MRSA) strains [1]. In China, ST239-SCC*mec*III accounts for around 75% of observed HA-MRSA infections and has been identified as the predominant clone [2,3]. Suspected MRSA outbreaks are normally investigated using traditional molecular typing techniques, such as multilocus sequence typing (MLST), staphylococcal chromosome cassette *mec* (SCC*mec*), and pulsed-field gel electrophoresis (PFGE), but recent studies have shown that whole-genome sequencing (WGS) has superior discriminatory power [4,5]. Success in tracking outbreaks [6,7,8] has demonstrated that the high resolution of WGS facilitates a better understanding of pathogens. In clinical microbiology, WGS has been used in different areas such as evolutionary studies, outbreak investigations [9,10], and phylogeographic distribution [6,11]. In this study, we applied traditional typing techniques and WGS to investigate a suspected outbreak of MRSA in the Neurosurgery Department at the Affiliated People’s Hospital of Jiangsu University in China.

## Materials and Methods

### Bacterial identification and drug susceptibility testing

A collection of 20 clinical isolates of MRSA was used in this study. 12 clinical isolates were recovered from lower respiratory tract specimens of patients. Direct environmental cultures were performed from different locations of the room with culture of inanimate surfaces performed with a 10 cm^2^ staphylococcus culture [12]. Eight environmental isolates were recovered as follows: four were from bedrails, two were from the surface of infusion pumps, one was from the working clothes of a healthcare worker, and the last was from the hands of a healthcare worker. Participation was voluntary and all participants signed an informed consent form prior to their inclusion in this study. No patient information is disclosed in this manuscript. The study was approved by the Ethics Committee of the Affiliated Hospital of Jiangsu University [no.2014030]. Bacterial identification was achieved by means of morphologic evaluation, Gram staining, and coagulase tests. The MRSA isolates were detected by several methods, including minimum inhibitory concentration of oxacillin (OX) and *mec*A detection by the polymerase chain reaction (PCR). We performed antimicrobial susceptibility testing using a VITEK-2 compact system (bioMérieux, Marcy l'Etoile, France) for the following drugs: OX, GEN, SXT, ERY, CLIN, TET, RIF, CIP, and VAN. Methicillin-sensitive *S*. *aureus* ATCC 29213 and MRSA ATCC 43300 were used as negative and positive controls, respectively, in each run. Results were interpreted according to the Clinical and Laboratory Standards Institute guidelines.

### Molecular typing

PCR was carried out for MLST and sequencing of the internal fragments of seven housekeeping genes was carried out to determine the allelic profile as previously described [13]. Strains were assigned to a sequence type (ST) using the MLST database (http://saureus.mlst.net/). The SCC*mec* type was determined using multiplex-PCR with previously reported primers [14]. Relatedness was determined using PFGE based on a previously described protocol [15]. The reference standard *Salmonella* Braenderup H9812 was used as the PFGE marker. Banding patterns were analyzed using BioNumerics software Version 6.0 (Applied Maths, Ghent, Belgium). A dendrogram was generated with the unweighted pair group method using average linkages based on Dice coefficients. To define a clonal group, as previously described, a similarity coefficient of 85% in combination with the criteria of clonal relatedness from Tenover et al. [16] was used.

### DNA sequencing

Genomic DNA from each MRSA isolate was extracted with an AxyPrep Bacterial Genomic DNA Miniprep Kit (Axygen Scientific, Inc., CA, USA) following the manufacturer’s instructions. WGS was performed on the Illumina MiSeq platform (250 PE, Illumina) using the paired-end mode (2×250) by Shanghai Personalbio Biotechnology (Shanghai, China), and the entire data set was provided by them for analysis. The sequencing data was aligned to the reference genome T0131 (NC_017347.1) to identify single-nucleotide polymorphisms (SNPs) as well as regions with insertions or deletions. This reference isolate was defined by means of MLST as ST239, the most common hospital-associated MRSA clone in Asian countries. SNPs in the core genome were identified using altered SNP-filtering parameters with GATK [17].

### Phylogenetic Analysis

Phylogenetic analysis was performed for the core genome SNPs of the 6 isolates in the present study and 3 references, including T0131, TW20 (NC_017331, NC_017332, and NC_017352) and JDK6008 (GenBank: CP002120), while FPR3757 USA300 (GenBank: CP000255) was included as an outgroup. Sequence alignment was performed using CLUSTALW2.0 with the default settings. A phylogenetic tree with 1000 bootstrap resamples of the alignment data sets was generated using the neighbor-joining method in MEGA5.0 [18]. The accessory genomes were excluded in the phylogenetic analysis because their variation may have arisen through horizontal gene transfer from unrelated lineages. These SNPs had an uneven distribution across the genome (S1, S2, S3, S4, S5 and S6 Figs), largely related to whether the SNP resided in the core or accessory regions. The accessory genomes, such as phages, transposons, or SCCmec, have an inherent potential for horizontal gene transfer between isolates, which could confound phylogenetic interpretation. Therefore, we distinguished between the “core” and “noncore” genome for subsequent analyses. Genes encoding potential antimicrobial resistance and various virulence genes were analyzed according to published data (S1 Table).

## Results

### Description of the outbreak

The Neurosurgery Department of the Affiliated People’s Hospital of Jiangsu University manages about 1700 patients per year, with 94 beds in three different wards, including two general wards and an intensive care unit (ICU). The ICU contains 14 beds in three large rooms and two single rooms. There are approximately 40 patients admitted to the ICU each month. MRSA was isolated during December 2012 and January 2013. Twelve samples were isolated from lower respiratory tract specimens. Environmental sampling was carried out, and eight isolates were recovered from bed rails, infusion pumps, overalls, and the hands of a healthcare worker. Patient SA13012 was the first who was found to acquire an MRSA infection in the Neurosurgical Department, and was subsequently transferred to the ICU. The last patient to have an infection was SA13005 (Table 1). Due to the identical antibiograms and PFGE patterns of 18 of the isolates, we selected four isolates, including SA13005, SA13007, and SA13012 from patients, and SA13023 from a doctor’s hands, for further analysis. The two remaining isolates, SA13002 and SA13009, with different antibiograms and PFGE patterns, were also sequenced. They were both isolated during the period of the outbreak in the Neurosurgery Department. The two patients also had a risk of infection with the bacteria. When the outbreak occurred, an investigation was triggered by the infection control team immediately and control measures were implemented. Meanwhile, all patients with known MRSA infections and colonization were transferred to isolation wards, and healthcare workers were assigned to specific patients to avoid cross-transmission. Newly admitted patients were isolated until their MRSA-carrier status was determined. Staff and family members were instructed to practice hand disinfection and wear gloves, gowns, and masks during any contact with a patient with an MRSA infection or with any potential fomites. All surfaces in the wards and offices were deep cleaned and disinfected using a chlorine-containing disinfectant with complete wiping of surfaces, and disinfection was implemented immediately if an area was polluted by a patient’s blood or body fluid. Clothes were changed and washed once every day. Patients and healthcare workers were screened using nasal and pharyngeal swabs, and the environment was also screened using swabs. Vancomycin was administered to control the infection. With these comprehensive interventions, we successfully halted the MRSA outbreak in the Neurosurgery Department within a short time. During this period, no patient died due to an MRSA infection. Afterwards, there were no new cases of MRSA during the next 2 months of surveillance. Furthermore, we detected three *S*. *aureus* isolates in the Neurosurgery ward, one of which was MRSA with resistance to fewer classes of antimicrobial agents, 2 months before the outbreak occurred.

**Table 1:** Distribution of the outbreak strains and onset time.

	12.19	12.21	12.22	12.25	12.27	12.31	1.4	1.6
**ICU**		SA13007(C10)	SA13009(C13)		SA13002(C03)	SA13001(C01)	SA13005(C09)	
			SA13006(C11)			SA13001(C12)		
**General wards**	SA13012(13)		SA13011(03)	SA13014(27)				
			SA13013(06)					
			SA13015(41)					
**Environment**								SA13016(infusion pumps)
								SA13017(bed rails)
								SA13018(bed rails)
								SA13019(bed rails)
								SA13020 (overalls)
								SA13021(infusion pumps)
								SA13022(bed rails)
								SA13023(doctor’s hands)

### Genetic typing

**Fig 1:**
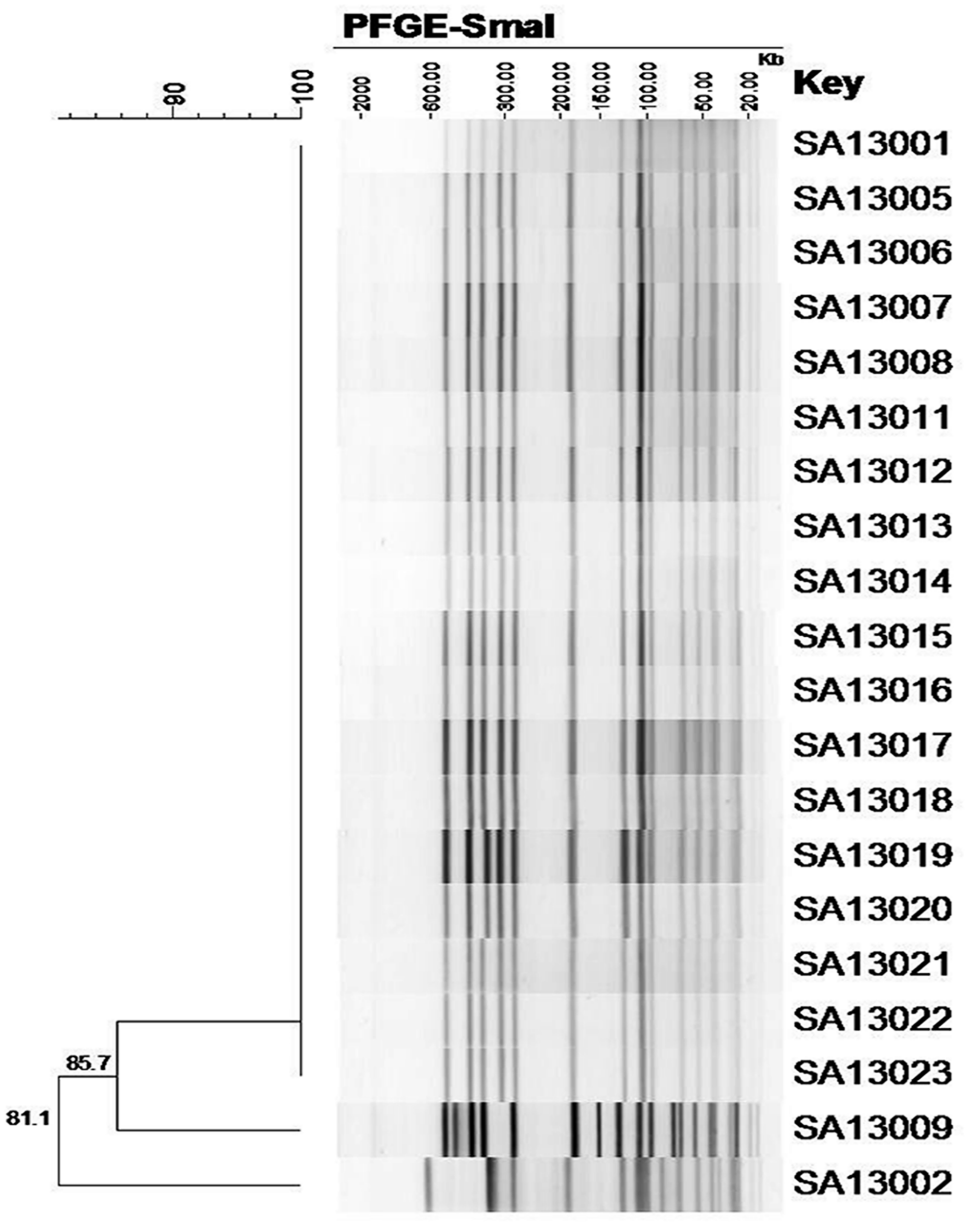
Dendrogram (% similarity coefficient) of the electrophoretic patterns of DNA macrorestriction with *Sma*I for the 20 MRSA isolates.

Among the 20 MRSA isolates, 18 had an identical PFGE profile (100% similarity coefficient). The remaining two isolates (SA13002 and SA13009) had distinct PFGE patterns with similarity coefficients of 81.1% and 85.7%, respectively, of which SA13002 did not meet the definition and was thus identified as unrelated (Fig 1). Each of the 20 outbreak isolates were identified as SCC*mec*III and belonged to clone ST239 in MLST.

### Use of whole-genome sequencing to define the diversity of dominant MRSA clones during the outbreak

**Fig 2:**
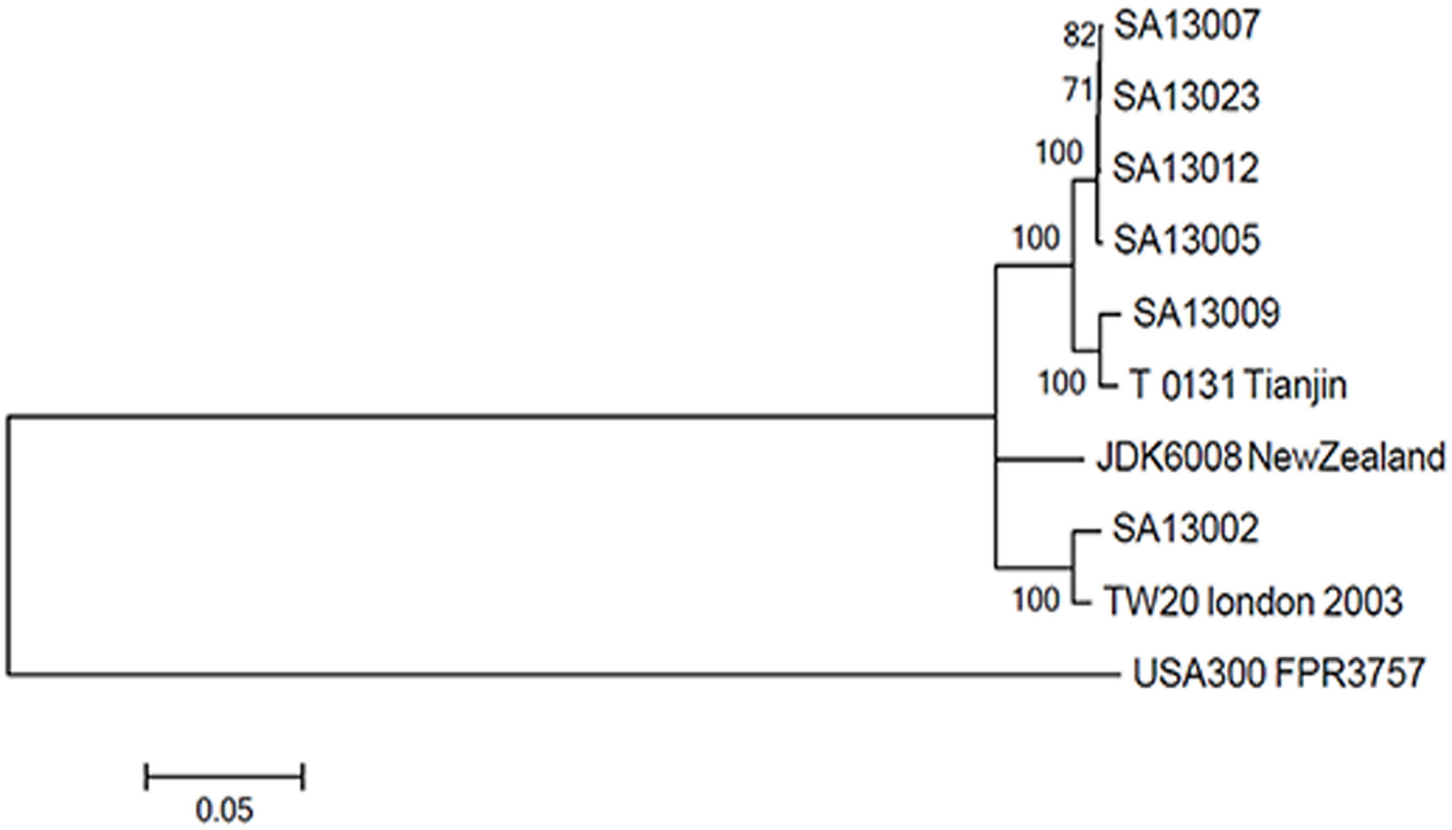
Phylogenetic analysis of the six outbreak strains. A phylogenetic tree was constructed from multiple alignment of the core genome SNPs of the 6 isolates in the present study and another 4 previous isolates from GenBank, where FPR3757 USA300 was included as an outgroup. A phylogenetic tree with 1000 bootstrap resamples of the alignment data sets was generated using the neighbor-joining method in MEGA5.0 with the contribution model of "Kimura 2-parameter". Bootstrap values are indicated at the nodes. The scale bar indicates the number of substitutions per position for a unit branch length.

Six isolates from patients and the ward environment were sequenced. We mapped the genome sequences of the six isolates against the reference sequence for ST239. Through a comparison with the published complete genome of T0131, the total number of SNPs and genome sequencing details are shown in S2 Table. The SNPs were relatively evenly distributed, suggesting that the majority of variants were due to point mutations rather than recombination (S1, S2, S3, S4, S5, and S6 Figs. Phylogenetic analysis revealed the presence of two distinct groups (Fig 2). One group contained four isolates associated with the Neurosurgery Department outbreak and SA13009 clustered with T0131 from Tianjin, while the other contained TW20 from London together with SA13002. Not more than 16 SNPs differentiated the four isolates from each other, including SA13005, SA13007, SA13012, and SA13023, compared with a mean of 997 SNPs between SA13002 and the four isolates mentioned (S2 Table). In addition, SA13009 differed from the outbreak strains at least 122 SNPs, suggesting that SA13002 and SA13009 were distinct from the outbreak strains. These observations were consistent with the results from the antibiograms.

### Resistance and virulence genes

Mechanisms of resistance to antibiotics arise either by mutations in the existing genome or acquisition of a specific gene. In this study, we detected various resistance genes and virulence genes using WGS data to determine antimicrobial resistance as well as virulence. Point mutations related to genes encoding potential antimicrobial resistance acquired by *S*. *aureus* were investigated in the sequenced genomes. The results in Table 2 show an almost complete concordance between the genotypes and phenotypes derived in the antibiogram. We also successfully detected various virulence genes associated with HA-MRSA based on WGS. Virulence genes, including *hla*, *pvl*, *psmα*, *psm-mec*, *sasX*, *sea*, *see*, and *tst-1*, were positive in all six isolates.

**Table 2:** Resistance and virulence of six outbreak strains.

Sample	OX	GEN	SXT	ERY	CLIN	TET	RIF	CIP	ST	SCC*mec*	Resistance genes	Toxin genes
SA13002	R	R	R	R	R	R	S	R	239	III	*mecA*	*hla*
											*tetK*	*pvl*
											*aacA-aphD*	*psmα*
											*dfrG*	*psm-mec*
												*sasX*
												*sea*
												*see*
												*tst-1*
SA13005	R	R	R	R	R	R	R	R	239	III	*mecA*	*hla*
											*tetK*	*pvl*
											*aacA-aphD*	*psmα*
											*dfrG*	*psm-mec*
												*sasX*
												*sea*
												*see*
												*tst-1*
SA13007	R	R	R	R	R	R	R	R	239	III	*mecA*	*hla*
											*tetK*	*pvl*
											*aacA-aphD*	*psmα*
											*dfrG*	*psm-mec*
												*sasX*
												*sea*
												*see*
												*tst-1*
SA13009	R	R	S	S	S	R	R	R	239	III	*mecA*	*hla*
											*tetK*	*pvl*
											*aacA-aphD*	*psmα*
											*dfrG*	*psm-mec*
												*sasX*
												*sea*
												*see*
												*tst-1*
SA13012	R	R	R	R	R	R	R	R	239	III	*mecA*	*hla*
											*tetK*	*pvl*
											*aacA-aphD*	*psmα*
											*dfrG*	*psm-mec*
												*sasX*
												*sea*
												*see*
												*tst-1*
SA13023	R	R	R	R	R	R	R	R	239	III	*mecA*	*hla*
											*tetK*	*pvl*
											*aacA-aphD*	*psmα*
											*dfrG*	*psm-mec*
												*sasX*
												*sea*
												*see*
												*tst-1*

## Discussion

*S*. *aureus* has a markedly clonal population structure [19,20,21,22]. Most disease-causing isolates belong to a small number of lineages or clonal complexes. Analysis of SNPs provides a means of determining the relatedness between epidemiologically linked isolates and of tracking bacterial evolution [23,24]. The estimated rate of core genome divergence provides sufficient diversity to separate recent from distant transmission events, and thereby dramatically improves contact tracing in endemic or outbreak settings. Consequently, the ability of WGS to discriminate closely related strains and track the evolution of clonal isolates offers the possibility of tracing transmission and identifying outbreaks [25]. Combining WGS with traditional molecular typing approaches, investigators have identified various outbreaks of bacterial agents [6,26,27].

In this study, molecular epidemiological data showed that 18 of the MRSA isolates belonged to be the same clone, with SA13009 closely related to this clone according to its PFGE profile, while SA13002 represented a different clone. SCC*mec* typing revealed that all outbreak isolates belonged to the SCC*mec*III lineage. This was in agreement with previous studies which showed that SCC*mec*III-positive isolates were the predominant multidrug-resistance type in Asian countries [28]. In addition, studies have demonstrated that two major MRSA clones are prevalent in Asian countries. The ST239-MRSA-III clone and its close relatives account for at least 90% of all HA-MRSA isolates in Asian countries [3,28]. Our results indicate that MRSA belonged to clone ST239, harboring SCC*mec*III and the virulence potential to cause an epidemic in the hospital environment.

WGS was used to successfully distinguish between outbreak and non-outbreak isolates by reconstructing a phylogenetic tree. The non-outbreak isolates of SA13002 and SA13009 differed from the outbreak isolates by at least 997 and 122 SNPs respectively, while the other four isolates differed from each other by not more than 16 SNPs. To avoid underestimating the frequency of patient-to-patient transmission, a SNP difference of >40 was used to exclude a recent transmission which was observed by Golubchik *et al* [29], while Uhlemann *et al* datermined 23 SNPs as the maximum pair-wise distance between pairs of isolates with clearly established epidemiological links [30]. Using SNP distances as a measure of genetic relatedness, together with epidemiological data, the antibiograms and conventional molecular typing methods, we believed that four admission isolates were highly related (<23SNPs), which suggesting a recent transmission, while excluding the possibility that SA13002 and SA13009 were direct transmission isolates [29,30,31]. Although SA13009 was more closely related to outbreak isolates than SA13002, we still believe it to be a non-outbreak isolates due to its different antibiogram, a very close percentage of PFGE to the 85% cut-off value used to define the same clone, and the SNP distances by WGS. The outbreak isolates were more closely related to T0131 than to TW20. This result was consistent with a previous study that found that the isolates in Beijing were more closely related to T0131, clustering closely with strains of the ‘Turkish clade’ using phylogenetic analysis [32]. T0131 has been reported to lack the φSP β-like prophage with the *sasX* gene characteristic of the Asian clade, while TW20 does contain these elements. In this study, we found that the outbreak strains were *sasX*-positive, in contrast to previously reported results. Our results reveal the distinct geographical spread of the ST239 lineage in our hospital and indicate possible transmission routes.

Researchers have used WGS to determine clinically relevant parameters significantly associated with toxicity and its implications on our understanding of virulence [33]. We found that the outbreak strains were positive for two virulence factors, *sasX* and *psm-mec*, which are crucial pathogenicity determinants for the colonization, dissemination, and pathogenicity of HA-MRSA [1,34] and are distributed in mobile genetic elements [32]. Studies have indicated that *pvl* also exists in HA-MRSA, while a portion of community-associated MRSA clones do not possess the *pvl* gene [35,36]. In this study, *pvl* was positive in all sequenced HA-MRSA samples. The *tst-1* gene was also detected in the genome, which could influence the outcome of the infection. Carriage of both *pvl* and *tst-1* has been reported in several studies with strains representing several different clonal backgrounds [37,38,39]. At this time, the clinical implications of harboring both toxins remains unclear, while the level of their expression may not be the same during infection [40]. Moreover, the *pvl*-carrying phage and *tst-1*-carrying pathogenicity island (SaPI) were both unique and could mobilize, suggesting that their emergence and dissemination may be forthcoming worldwide [40].

Resistance genes detected by sequencing, in comparison to phenotypic antibiograms, showed concordance with these findings, indicating that WGS can be used to guide treatment and discover new resistance mechanisms. Studies have demonstrated that WGS is a promising method to provide accurate predictions of antimicrobial resistance in *S*. *aureus* [41]. Using WGS, it was possible to sequence toxin genes associated with the pathogenicity of HA-MRSA [33,42], providing proof that WGS could immediately identify toxin genes and thereby predict the virulence of MRSA.

Molecular typing techniques have further indicated that the infection-control team of the hospital correctly identified an outbreak in the Neurosurgery Department using an antibiogram approach and successfully controlled it. However, SA13002 and SA13009 exhibited distinct antibiograms, had a different PFGE profile, and were identified as non-outbreak isolates genotypically. Importantly, antibiograms are not always informative for outbreak investigations. A previous study showed that two isolates of ST22 were unrelated, aided by phylogenetic analysis, despite the fact that the antibiogram for each was identical [10]. It is important to distinguish between outbreak and non-outbreak isolates, in that control measures can be implemented promptly to control an outbreak and unnecessary control measures can be avoided. WGS was also used to infer the most likely transmission route of the outbreak. Consistent with our observations of the presentation of infections chronologically, the first transmission of the outbreak isolate was predicted to go through patient SA13012, who presented with pulmonary symptoms earlier than the other patients. However, the outbreak source remains unknown because of the lack of longitudinal surveillance of clinical isolates before the outbreak. We also cannot determine when and how transmission occurred. Therefore, more work is needed in the future.

In conclusion, a suspected outbreak was confirmed by traditional methods and WGS. To prevent MRSA outbreaks in our hospital, special attention is required for the screening and treatment of MRSA infection. Furthermore, we highlight the potential of WGS in the analysis of hospital outbreaks and its application in infection control to prevent MRSA transmission, to predict resistance and virulence, and to search for new drugs for therapy. With advances in technology and reduced costs, we believe that WGS will be widely used in the analysis of pathogen-based outbreaks.

### Nucleotide sequence accession numbers

The nucleotide sequences of the outbreak strains analyzed in this study were deposited in the National Center for Biotechnology Information GenBank database under accession numbers SAMN03856026 and SAMN03878399 to SAMN03878403.

## Supporting Information

S1 Fig SNP distribution of SA13002 in the whole genome by using cgview mapping software. (PDF)

S2 Fig SNP distribution of SA13005 in the whole genome by using cgview mapping software. (PDF)

S3 Fig SNP distribution of SA13007 in the whole genome by using cgview mapping software. (PDF)

S4 Fig SNP distribution of SA13009 in the whole genome by using cgview mapping software. (PDF)

S5 Fig SNP distribution of SA13012 in the whole genome by using cgview mapping software. (PDF)

S6 Fig SNP distribution of SA13023 in the whole genome by using cgview mapping software. (PDF)

S1 Table Sequences used in the resistome and toxome pseudomolecules that were mapped with the MiSeq data. (DOCX)

S2 Table Summary results of mapping MiSeq data to the ST239 reference (T0131). (DOCX)
