# Supplementary material for: Whole-Genome Sequencing for the Investigation of a Hospital Outbreak of MRSA in China
Source: PLoS One. 2016 Mar 7;11(3):e0149844. doi: 10.1371/journal.pone.0149844 (PMC4780730; doi:10.1371/journal.pone.0149844)
Supplement: S6 Fig — (PDF) [file pone.0149844.s006.pdf]

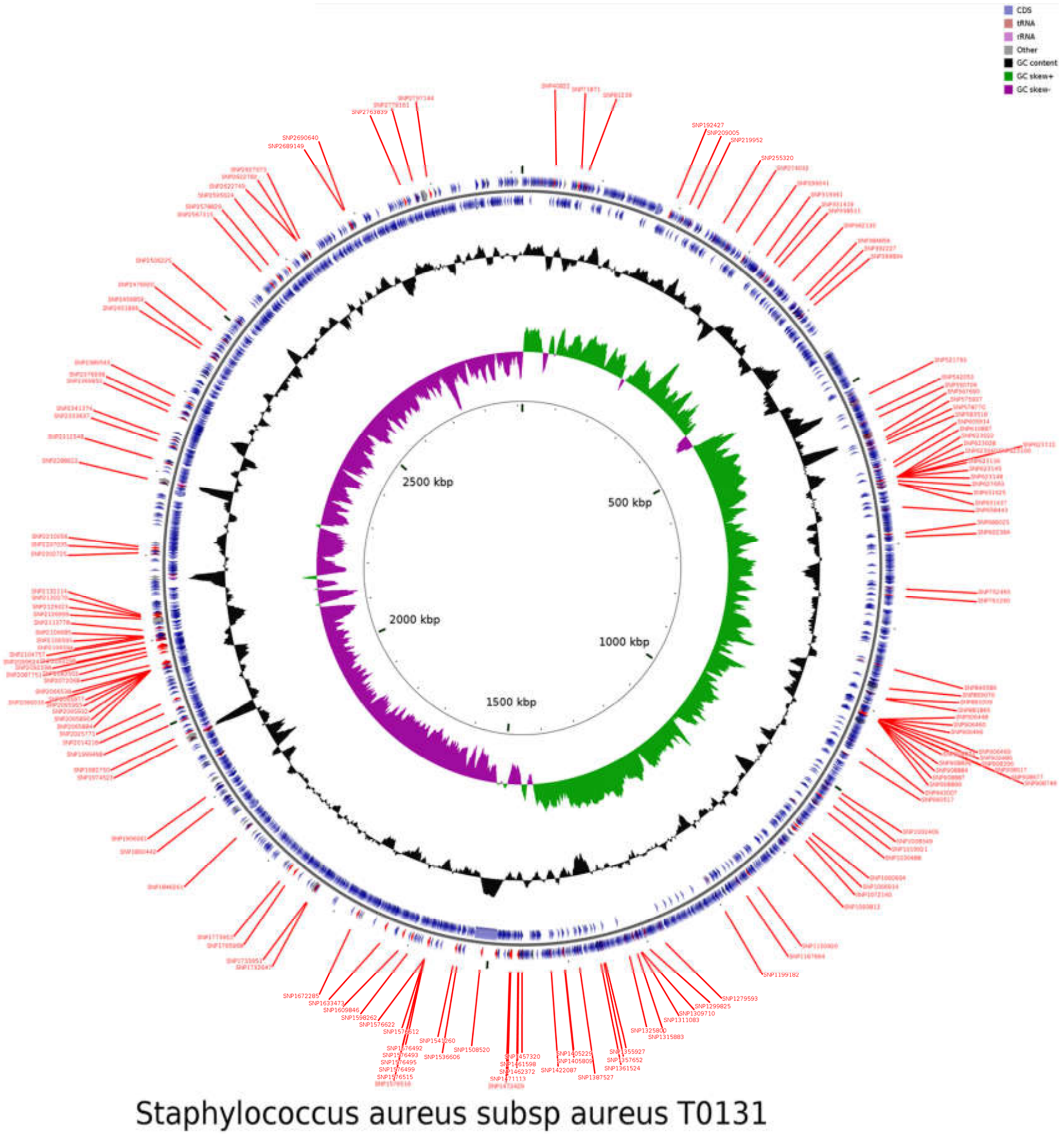

S6 Fig SNP distribution of SA13023 in the whole genome by using cgview mapping software.
