# Supplementary material for: Whole-Genome Sequencing for the Investigation of a Hospital Outbreak of MRSA in China
Source: PLoS One. 2016 Mar 7;11(3):e0149844. doi: 10.1371/journal.pone.0149844 (PMC4780730; doi:10.1371/journal.pone.0149844)
Supplement: S1 Table — (DOCX) [file pone.0149844.s007.docx]

**S1 Table Sequences used in the resistome and toxome pseudomolecules that were mapped with the MiSeq data.**

| **Gene** | **Accession number** |
| --- | --- |
| mecA  ermA  ermC  aacA-aphD  tetK  dfrG  SasX  psm α  psm-mec  hla  tst-1  lukS-PV  lukF-PV  sea  seb  sec  sed  see  eta  etb | X52592  P06699  P13978  P14507  P02983  C7C2U7  SATW20­_21850  BK006301.1  AB729111.1  NC_007795.1  J02615  X72700  X72700  M18970  M11118  X05815  M28521  M21319  P09331  AAA26628 |
