# Supplementary material for: Whole-Genome Sequencing for the Investigation of a Hospital Outbreak of MRSA in China
Source: PLoS One. 2016 Mar 7;11(3):e0149844. doi: 10.1371/journal.pone.0149844 (PMC4780730; doi:10.1371/journal.pone.0149844)
Supplement: S2 Table — (DOCX) [file pone.0149844.s008.docx]

**S2 Table Summary results of mapping MiSeq data to the ST239 reference (T0131).**

| **Isolate ID** | **ST** | **Total no. of reads** | **Mapped Reads** | **Mapped**  **%** | **Coverage (×)^1^** | **Number of SNPs^2^** | **Number of INDELs** | **Number of CNV** |
| --- | --- | --- | --- | --- | --- | --- | --- | --- |
| SA13002  SA13005  SA13007  SA13009  SA13012  SA13023 | ST239  ST239  ST239  ST239  ST239  ST239 | 1,958,503 1,631,172  1,963,819 1,641,803  1,872,204 1,468,571 | 1,593,095 1,581,170  1,908,490 1,591,058  1,808,590 1,419,434 | 81.34  96.93  97.18  96.91  96.6  96.65 | 156  130  156  131  149  117 | 1188  253  260  122  250  244 | 132  62  60  41  56  62 | 30  7  7  7  9  6 |
